# First evidence of TRPV5 and TRPV6 channels in human parathyroid glands: possible involvement in neoplastic transformation

**DOI:** 10.1111/jcmm.12372

**Published:** 2014-08-28

**Authors:** Laura Giusti, Filomena Cetani, Ylenia Da Valle, Elena Pardi, Federica Ciregia, Elena Donadio, Claudia Gargini, Ilaria Piano, Simona Borsari, Ali Jaber, Antonella Caputo, Fulvio Basolo, Gino Giannaccini, Claudio Marcocci, Antonio Lucacchini

**Affiliations:** a Department of Pharmacy, University of PisaPisa, Italy; b Department of Clinical and Experimental Medicine, University of PisaPisa, Italy; c Department of Surgical, Medical, Molecular Pathology and Critical Area, University of PisaPisa, Italy

**Keywords:** TRPV5, TRPV6, parathyroid glands, calcium, adenoma

## Abstract

The parathyroid glands play an overall regulatory role in the systemic calcium (Ca^2+^) homeostasis. The purpose of the present study was to demonstrate the presence of the Ca^2+^ channels transient receptor potential vanilloid (TRPV) 5 and TRPV6 in human parathyroid glands. Semi-quantitative and quantitative PCR was carried out to evaluate the presence of TRPV5 and TRPV6 mRNAs in sporadic parathyroid adenomas and normal parathyroid glands. Western blot and immunocytochemical assays were used to assess protein expression, cellular localization and time expression in primary cultures from human parathyroid adenoma. TRPV5 and TRPV6 transcripts were then identified both in normal and pathological tissues. Predominant immunoreactive bands were detected at 75–80 kD for both vanilloid channels. These channels co-localized with the calcium-sensing receptor (CASR) on the membrane surface, but immunoreactivity was also detected in the cytosol and around the nuclei. Our data showed that western blotting recorded an increase of protein expression of both channels in adenoma samples compared with normal glands suggesting a potential relation with the cell calcium signalling pathway and the pathological processes of these glands.

## Introduction

Transient receptor potential vanilloid calcium channels (TRPV) constitute one of the seven subfamilies of the large and functionally adaptable superfamily of cation channels named transient receptor potential (TRP) 1,2. Among all members of TRPV family, TRPV5 and TRPV6, which are highly Ca^2+^-selective channels, play a pivotal role in the Ca^2+^-reabsorption in the kidney and intestine respectively 3,4. The biological activity of TRPV5 and TRPV6 is coordinated and regulated in a different manner by various regulatory mechanisms, such as calciotropic hormones [1,25-dihydroxyvitamin D and parathyroid hormone (PTH)], pH, internal Ca^2+^ concentrations, and interaction with regulatory proteins 5,6. Besides the maintenance of Ca^2+^ balance in healthy tissues, overexpression of TRPV5/TRPV6 mRNA and proteins was observed in advanced stages of prostate, colon, breast, thyroid and ovarian carcinomas and leukaemia. However, little is known about their role in initiation or progression of tumours 7–10, although high expression of calcium channels is characteristic of cancer diseases through the control of processes, such as proliferation and apoptosis resistance 11,12.

As mentioned above, a concerted action of associated proteins in the regulation of the activity of TRPV5 and TRPV6 has been reported 5,6, in particular inhibitor of protein B-box, SPRY-domain containing protein (BSPRY) 5,13 and RGS2 14 or positive regulator proteins as klotho 15,16 and WNK3 17. In a previous work, by using a proteomic approach, we examined the global changes of protein profile between parathyroid adenomas and normal parathyroid tissues in an attempt to identify key proteins involved in neoplastic transformation 18. Among signal transduction proteins, we observed a significant decrease of BSPRY expression in parathyroid adenomas. Taking into account the regulatory role of the BSPRY and the involvement in cell proliferation of TRPV5/TRPV6, we investigated the presence of both channels in parathyroid adenoma and in parathyroid normal gland, either at mRNA or protein level. In the present study, for the first time, we provide evidence of the presence of TRPV5 and TRPV6 in human parathyroid glands and their protein overexpression in the parathyroid adenomas.

## Methods

### Tissue specimens

Ten parathyroid adenomas from patients with sporadic primary hyperparathyroidism (PHPT) and three normal parathyroid glands obtained from normocalcaemic patients operated for nodular goitre were included in the study. All tissues were immediately snap frozen in liquid nitrogen after surgery and stored at −80°C until use. Histopathological classification was according to the World Health Organization's (WHO) guidelines 19. All patients gave their informed consent. The study was approved by the local Ethics Committee.

### Protein extraction

Protein extraction was obtained essentially as previously described 18. Briefly, 20–30 mg of tissue sample was suspended in 20 volumes of 10 mM Tris-HCl pH 7.4 containing a protease inhibitor cocktail (Sigma-Aldrich, St Louis, MO, USA), and homogenized on ice with a potter homogenizer. The homogenate was centrifuged at 14,000 × g in an eppendorf centrifuge at 4°C for 30 min. and the resulting supernatant was immediately stored at −80°C (S1) while the pellet (membrane fraction, P1) was resuspended in rehydration solution (7 M Urea, 2 M thiourea, 4% CHAPS, 60 mM DTT, 0.002% bromophenol blue) and incubated for 45 min. under rotation at 4°C. After incubation, the sample was centrifuged for 10 min. at 14,000 × g to remove undissolved material. The protein concentrations were measured with an RC-DC Protein Assay from Bio-Rad (Hercules, CA, USA), by using bovine serum albumin as a standard.

### Western blot analysis

Western blot analyses were carried out on protein extracts (P1 and S1) as previously described 18,20. Aliquots of 60 μg of proteins were mixed with SDS sample buffer (Laemmli solution) and heated at 100°C for 5 min. Proteins were run on 8% SDS-PAGE gels, and transferred onto nitrocellulose membranes (0.2 μm) by using 100 V for 30 min. (Tetra cell apparatus, Bio-Rad). Anti-TRPV5 and anti-TRPV6 goat polyclonal antibodies (K-17 and L-15, respectively, Santa Cruz Biotechnology Inc., Santa Cruz, CA, USA) were used at dilution of 1:200. After incubation with appropriate secondary antibody, immunoreactions were visualized by using ECL detection system. The chemiluminescent images were acquired by LAS4010 (GE Health Care Europe, Uppsala, Sweden).

### Semi-quantitative and quantitative PCR

RNA extraction was performed with the PureLink RNA Mini Kit, according to the manufacturer's instructions (Invitrogen, Life Technologies Ltd, Paisley, UK). The concentration and purity of all RNA samples, eluted in a final volume of 30 μl RNase-Free water, were determined with a Nano-Drop® ND-1000 UV-Vis Spectrophotometer (NanoDrop Technologies, Wilmington, DE, USA). One microgram of total RNA for each sample was reverse transcribed into cDNA in a 20 μl reaction volume by using Superscript III reverse transcriptase (Invitrogen, Life Technologies Ltd). Qualitative PCR reactions were carried out in a MJ Research thermal cycler. TRPV5 and TRPV6 primers and PCR conditions were derived from Hoenderop *et al*. 21. cDNA yields for genes of interest were compared with glucose-6-phosphate dehydrogenase (G6PD) as a housekeeping control gene. Amplified TRPV5 and TRPV6 cDNAs were subsequently separated on 1.5% agarose gel, stained with ethidium bromide and photographed under UV illumination. The photographs were scanned on densitometry (Bio-Rad Laboratories) and band intensity was evaluated by using Quantity One software (Bio-Rad Laboratories). Each sample value was normalized for loading errors (dividing by intensity of G6PD); data were expressed as arbitrary units (A.U.) representing the ratio between the intensities of the band of interest and of the band corresponding to the control protein. PCR bands were cut from gel and directly sequenced to confirm the identities of the two transcripts.

Quantitative gene expression study was performed by real-time PCR by using TaqMan Gene Expression Assays (Applied Biosystems, Foster City, CA, USA). The primer/probe mixes which were used to amplify TRPV5 and TRPV6 genes and the housekeeping gene glyceraldehyde-3-phosphate dehydrogenase (GAPDH) were both purchased from Applied Biosystems (TaqMan® GeneExpression Assays-on-Demand). Briefly, PCR reaction was carried out in 96-well optical reaction plates by using cDNA equivalent to 20 ng total RNA for each sample (parathyroid adenomas and a pool of 3 normal parathyroid glands) in a volume of 25 μl by using the TaqMan Universal PCR Master Mix (Applied Biosystems, Life Technologies Ltd) according to the manufacturer's instructions. PCR was run on the ABI Prism 7700 Sequence Detector (Applied Biosystems, Life Technologies Ltd). The analysis of relative gene expression data was performed with the ΔΔCT method with the housekeeping gene GAPDH as an endogenous control/reference assay. The results were expressed as the amount of target gene normalized to the endogenous reference and relative to a calibrator (pool of normal parathyroid tissues).

### Cell culture

Specimens of four different adenoma parathyroid glands from patients with sporadic PHPT were used for cell culture experiments. Primary cell cultures were prepared directly after parathyroidectomy (PTx) according to published procedures 22. Briefly, the excised parathyroid glands were immediately suspended in sterile medium after PTx and minced with scalpels after the removal of visible fat and connective tissue, and cell suspensions were prepared by incubation in 2 mg/ml collagenase dissolved in DMEM-F12 medium, for 2 hrs at 37°C. Then, the cells were mechanically dispersed, filtered through sterile gauzes and centrifuged. Washed pellets were suspended in DMEM-F12 medium added with 10% foetal bovine serum. The cells were counted and plated at a density of 5 × 10^4^ cells/well onto the BioCoat Cellware 8 wells chamber slide pre-coated with Poly-Lysine (BD Biosciences, Franklin Lakes, NJ, USA), grown at 37°C in 5% CO_2_ humidified atmosphere for 24, 48 and 72 hrs. At these time-points, PTH release from plated parathyroid chief cells was quantified. Briefly, medium was removed from dishes by aspiration and diluted 1:100 in fresh culture medium. iPTH(1–84) concentrations were, immediately or after −20°C freezing, measured by an immunochemiluminometric assay (Liaison analyzer, Diasorin, Stillwater, MN, USA). Each sample was tested in triplicate.

### Immunocytochemistry

Primary human parathyroid cells incubated for 24, 48 and 72 hrs were fixed in 2% paraformaldehyde in 0.1 M phosphate buffer, washed three times in PBS, rinsed and blocked for 45 min. in a solution of 0.1% Triton-X 100, 1% bovine serum albumin (BSA) in PBS. After washing, the cells were incubated with goat polyclonal anti-TRPV5 or anti-TRPV6 primary antibodies (1:250; Santa Cruz Biotechnology) or mouse monoclonal anti-CASR primary antibody (1:500; Affinity BioReagents, Suite, CO, USA) with 0.03% Triton-X 100 and 1% BSA. The co-localization was obtained by incubation of anti-TRPV5 and anti-CASR or anti-TRPV6 and anti-CASR. The primary antibodies were incubated overnight at 4°C. After the washing, to visualize single staining, the cells were incubated with donkey anti-goat antibody (1:400; Sigma-Aldrich) or with goat anti-mouse (1:400; Invitrogen Life Technologies Ltd) conjugated with Alexa Fluor 488; furthermore, to visualize the co-localization, the cells were incubated with donkey anti-goat conjugated with Alexa Fluor 488 (1:400; Sigma-Aldrich) and with donkey anti-rabbit conjugated with Rhodamin-RedX (1:400; Jackson ImmunoResearch Laboratories, Suffolk, England, UK). The secondary antibodies were incubated for 2 hrs at room temperature and then the slides were covered with Vectashield (Vector Laboratories, Burlingame, CA, USA).

The immunofluorescence staining images of TRPV5 and TRPV6 were detected by a confocal laser scanning microscope (Leica TCS-SP5) by using different 40× zoom 1 or 2 (see legends of figures) oil objective with 1.45 NA and a recommended pinhole size of less than 1.0 micrometre (to estimate the cell size, see scale bars on the figures). The scanning fields were 250 × 250 μm in size and consisted of 4 consecutive acquisitions along the *z*-axis, totally encompassing a thickness of 4 μm. Saved files were focus extended images obtained automatically by superposition of the four focal planes. The images were processed with PhotoshopCS3 software.

### Surface biotinylation of parathyroid adenoma cells

Cell-surface biotinylation was carried out by using Cell Surface Protein Isolation Kit (Thermo Scientific, Rockford, IL, USA) according to the manufacturer's instructions. Briefly, cell flasks were rinsed twice with 5 ml of ice-cold PBS, then surface proteins were biotinylated by incubating cells with 4 mg/ml sulfo-NHS-SS-Biotin in PBS for 30 min. with horizontal shaking at 4°C. After labelling, the flasks were washed with quenching buffer for 10 min. at 4°C and then the cells were gently scraped and the contents transferred in a conical tube and centrifuged at 500 × g for 5 min. The pellet was rinsed three times with 5 ml of 0.025 M Tris, 0.15 M NaCl pH 7.2 and cells were finally lysed in lysis buffer for 30 min. on ice. Cell lysates were centrifuged at 10,000 × g for 5 min. at 4°C and supernatants were equilibrated for 60 min. with neutravidin-agarose beads at 4°C with end-over-end mixing by using a rotator. Beads were washed three times and biotinylated surface proteins were then released by the addition of Laemmli solution. Control samples were obtained incubating the cells without sulfo-NHS-SS-Biotin addition. Western blots were performed as described above. The experiment was performed in duplicate.

### Data analysis and statistics

The antigen-specific bands were quantified by using the ImageQuant-L (GE Health Care Europe Uppsala, Sweden). The significance of the differences (*P* ≤ 0.05) between adenoma and normal samples was calculated by the Student's *t*-test. All the experiments were performed in duplicate.

In the gene expression experiments, the fold of difference between normal and adenoma parathyroid samples was calculated as 2^−ΔΔCt^, where ΔΔCt = (ΔCt sample − ΔCt calibrator) and ΔCt = (Ct value of TRPV5 or TRPV6 − Ct value of GAPDH). The average Ct value of a pool of normal parathyroids was used as the calibrator in the analysis. Each experiment was performed in duplicate. The Student's *t*-test was used to test the significance of differential mRNA expression between samples, while the risk level (*P*) was set at *<0.05, **<0.01, ***<0.001.

## Results

### Western blot and Cell-surface presence of TRPV5 and TRPV6

**Figure 1:**
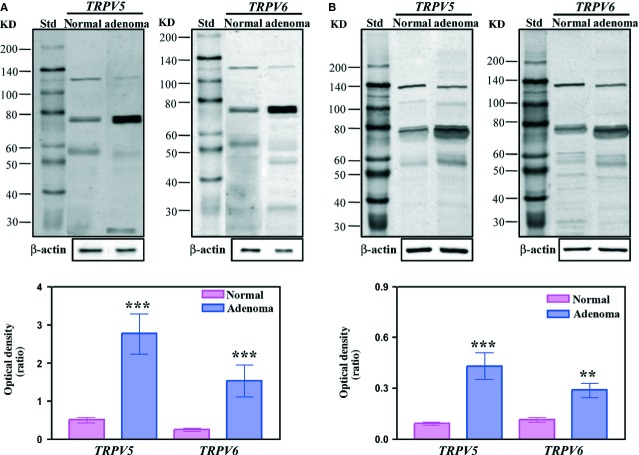
Representative immunoblot of protein membrane extracts (P1) and soluble fraction (S1) from normal and adenoma parathyroid samples. Aliquots of normal and adenoma parathyroid P1 (**A**) and S1 (**B**) samples (60 μg of proteins) were run by using 8% resolving capacity. Proteins were transferred onto nitrocellulose membranes and incubated with specific antibodies against the target proteins (TRPV5 and TRPV6). Below the immunoblots the corresponding densitometry of the blots are shown for 75–80 kD bands. Bar graphs represented the mean of the normal (*n* = 3) and adenoma (*n* = 10) P1 and S1 samples. Statistically significant differences were determined by Student's *t*-test and *P*-values calculated (**P < 0.01; ***P < 0.001).

**Figure 2:**
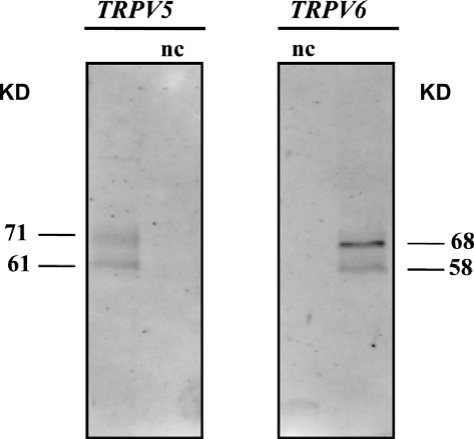
Surface biotinylation of plasma membranes of primary human parathyroid cells. Representative immunoblot analysis of surface-biotinylated proteins precipitated with neutravidin-agarose beads and probed with anti-TRPV5 and anti-TRPV6 antibodies, which revealed human TRPV5 and TRPV6 detection. Specificity was confirmed by negative control (nc).

Western blot analysis was performed to determine the presence of TRPV5 and TRPV6 proteins in parathyroid tissues. The search was carried out both in membrane extracts (P1) and soluble fraction (S1). Figure1A and B show an example of western blots of P1 and S1 samples, respectively, for both TRPV5 and TRPV6. The prominent immunoreactive band was detected at about 75–80 kD for both TRPV proteins. Additional weaker bands at about 135, 110, 47–60 and 30 kD were observed in P1 (Fig.1A) and S1 samples (Fig.1B) with different patterns between TRPV5 and TRPV6. Moreover, a major number of reaction bands (probably proteolytic products) were observed for TRPV6 in S1 samples. The derived bar graphs for prominent band were also reported. After comparison of WB of adenoma (*n* = 10) and normal samples (*n* = 3), a significant increase of expression was detected for 75–80 kD band both in P1 (*P* < 0.001) and S1 (*P* < 0.01) adenoma extracts. Significant differences were also observed for 30 and 47–60 kD bands both in P1 and S1 samples with *P*-values ranging from 0.05 to 0.01. To confirm the presence of TRPV5 and TRPV6 within the plasma membrane, we utilized a surface biotinylation approach. TRPV5 and TRPV6 antibodies detected two bands with molecular weight ranging from 58 to 71 kD (Fig.2) after biotinylation of primary human parathyroid cells.

### Semi-quantitative PCR

**Figure 3:**
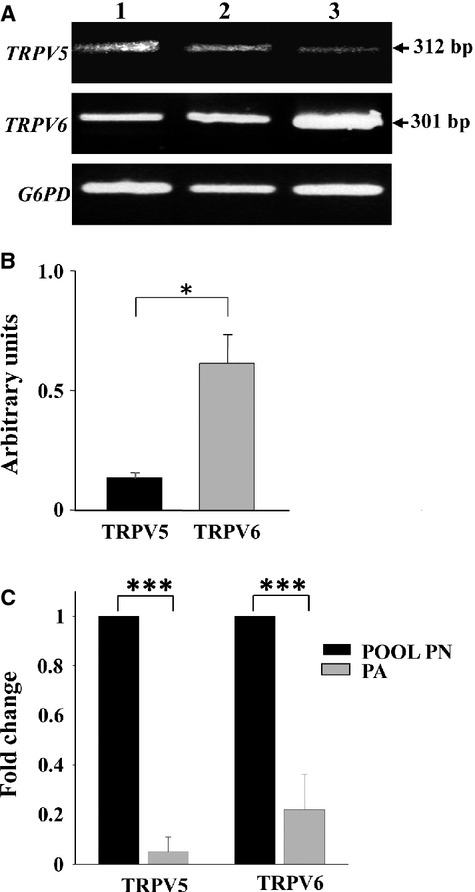
Semi-quantitative and quantitative PCR (qPCR) for TRPV5 and TRPV6 genes. (**A**) The PCR products of reverse trascripted mRNAs were separated by agarose gel electrophoresis and detected by ethidium bromide staining. The arrows shows the amplified TRPV5 and TRPV6 cDNAs with the 301 and 312 bp expected size, respectively, in two representative different parathyroid adenomas (lanes 1, 2) and in one human non-pathological pancreas used as positive control (lane 3). G6PD was used as loading control. (**B**) Bar graph representing quantified densitometry of data partially showed in (**A**). Each bar represents mean values of band density of 10 different parathyroid adenomas with positive error bars (SD). Electrophoresis separation was repeated twice. Each sample value was normalized dividing by the intensity of G6PD and the obtained data were expressed as arbitrary units. The graph shows the relative abundance of TRPV6 transcripts in parathyroid adenomas (PA) compared with TRPV5. (**C**) Bar graph representing real-time PCR results. The figure shows the mRNA expression fold change of the two target genes (TRPV5 and TRPV6) by using the 2ΔΔCT method relative to the internal control gene (GAPDH) in normal and pathological parathyroid glands. The fold difference was calculated as 2−ΔΔCt, where ΔΔCt = (ΔCt sample − ΔCt calibrator) and ΔCt = (Ct value of TRPV5 or TRPV6 − Ct value of GAPDH). The average Ct value of a pool of three normal parathyroids (PN) was used as the calibrator in the analysis. Genes up or down-regulation were expressed as mean ± SD of TRPV5 or TRPV6/GAPDH ratio of three independent experiments and a total of eight different parathyroid adenomas (PA) analysed in duplicate. Statistical significance was determined by Student's *t*-test where *P < 0.05, **P < 0.01 and ***P < 0.001.

Reverse transcriptase PCR analysis showed the presence of both TPRV5 and TPRV6 transcripts in parathyroid adenomas. Direct sequencing of the bands relative to TRPV5 and TRPV6 transcripts confirmed their identity (data not shown). The results of a representative experiment are shown in Figure3A. Densitometric data of bands obtained by two independent experiments revealed a statistically significant relative abundance of TRPV6 (*P* < 0.05) compared with TRPV5 transcripts in parathyroid adenomas (Fig.3B).

### Quantitative PCR

The comparative quantitative PCR showed that parathyroid adenomas had a lower level of expression with respect to the pool of normal parathyroid glands (*P* < 0.001), both for TRPV5 and TRPV6 genes, which was on average 16 and fourfold less respectively (Fig.3C).

### Immunocytochemistry

**Figure 4:**
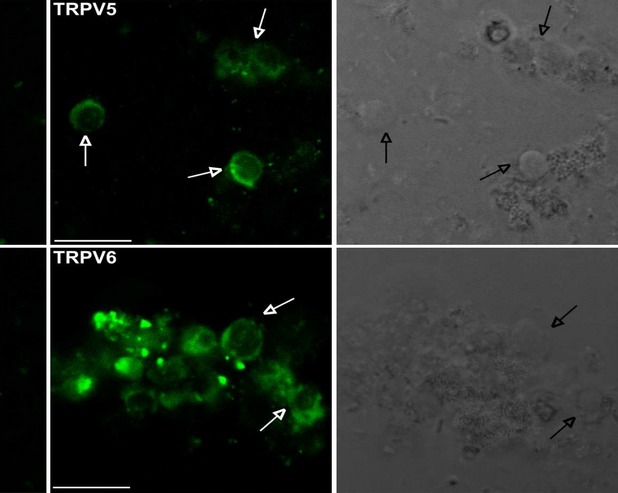
Confocal images of primary human parathyroid cells stained with antibody anti-TRPV5 and anti-TRPV6 (green, left panels). In the right panels the corresponding bright field images. Arrows indicate corresponding cell bodies. In the columns at the left-hand side are the 2nd antibody control images. The immunofluorescence staining images were detected by a confocal laser scanning microscope (Leica TCS-SP5) by using 40× zoom 2 oil objective with 1.45 NA and a recommended pinhole size of less than 1.0 μm; scale bars: 10 μm.

**Figure 5:**
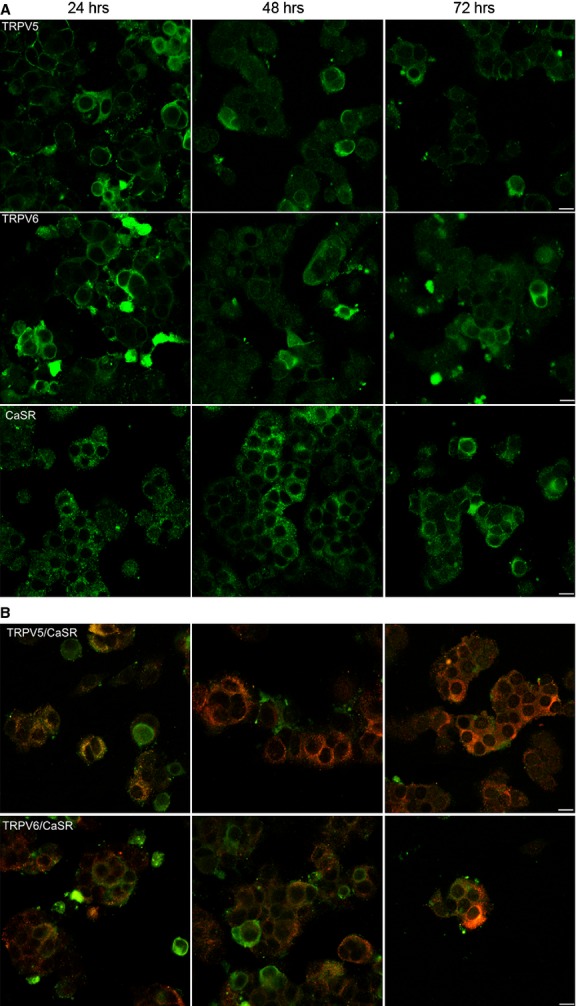
Confocal images of primary human parathyroid cells maintained at different times of incubation. (**A**) It shows the expression of TRPV5, TRPV6 and CASR (green), respectively, at 24, 48 and 72 hrs. (**B**) It shows the co-localization between TRPV5, TRPV6 (green) and CASR (red). The immunofluorescence staining images were detected by a confocal laser scanning microscope (Leica TCS-SP5) by using 40× zoom 1 oil objective with 1.45 NA and a recommended pinhole size of less than 1.0 μm; scale bars: 10 μm.

Primary human parathyroid cells maintained in culture, positive to PTH secretion measurements (30 ± 2.8 pg/ml/60 min./well), expressed both channels when assayed at 24, 48 and 72 hrs. Figure4 shows confocal images of parathyroid cells stained with antibody anti-TRPV5 and anti-TRPV6 (green, left panels). In the right panels are shown the corresponding bright field images. Both TRPV5 and TRPV6 channels are probably expressed in the cytosol and on the plasma membrane. The expression of TRPV5, TRPV6 and CASR as a function of the time of cells incubation is shown in Figure5A, where specific markers were employed. Figure5B shows the co-localization of TRPV5, TRPV6 (green) and CASR (red) in the majority of parathyroid cells.

## Discussion

TRPV5 and TRPV6 are two high selective Ca^2+^ channels predominantly localized in epithelial tissues and presumably involved in transcellular calcium transport 4. Their main physiological functions occur at kidney and intestine level where these channels are prime targets of calciotropic hormones, such as PTH and 1,25-dihydroxyvitamin D, in the control of Ca^2+^ reabsorption from pro-urine (TRPV5) and intestine Ca^2+^ uptake (TRPV6). Moreover, high expression of TRPV6 was described in murine 23 and human 24–26 placenta where it is a major pathway through which calcium is supplied to the foetus. These physiological functions are the result of a concerted action of associated proteins, which, in addition to Ca^2+^ itself, play key roles in the positive (*i.e*. klotho, WNK3) and negative (*i.e*. BSPRY) regulation of TRPV5 and TRPV6 channels 5,6,13–17. In a previous work, we demonstrated a significant down-regulation of BSPRY protein in adenoma with respect to normal glands 18. The identification of a negative modulator of TRPV5 and TRPV6 channels led us to investigate the presence of these channels in human parathyroid glands. Up to date, the presence of TRPV5 and TRPV6 has been demonstrated in various pathological and non-pathological human tissues and cell lines 7. The expression of CAT-L, a human homologue of rat TRPV6, has previously been investigated by Northern blot analysis in two healthy parathyroid tissues by Wissenbach *et al*., with negative results 27. In this work, the presence of TRPV5 and TRPV6 proteins and of their mRNAs was studied by using human parathyroid tissues, while their cellular localization and time expression were evaluated by immunocytochemical assay on primary human adenoma parathyroid cells.

Reverse transcriptase PCR, western blot and immunocytochemistry assays all indicate, for the very first time, that TRPV5 and TRPV6 channels are expressed in both human normal and pathological parathyroid glands. Quantitative evaluation of western blotting data showed an overexpression of both channels in adenoma with respect to normal parathyroid tissues, either for membrane or for soluble fraction preparations with different ratio. Immunoblot patterns besides main band corresponding to the core of protein showed other bands ranging from 85 to 130 kD probably resulted from glycosylation processes. Moreover, we suggested that a proteolytic effect might be responsible for detection of low molecular weight bands. High levels of these proteins in the soluble fraction suggest an active recycle of these channels in parathyroid glands, as already described 6,15,28,29. Moreover, immunocytochemical results confirmed that primary human parathyroid cells express TRPV5 and TRPV6 within the cytosol around nuclei and on the plasma membrane, while surface biotinylation demonstrated the presence of these channels in plasma membranes. As far as glycosylation is concerned, a modification might be responsible also for different observed bands with molecular weight ranging from 85 to 130 kD as previously suggested.

As mentioned above, it has been documented that the number of TRPV5 and TRPV6 on the plasma membrane is determined by counterbalance between insertion by forward trafficking from Golgi and removal by endocytosis to endosomes. This regulation appears operated by glycosydase enzymes, such as klotho, and leads to accumulation of channels on the plasma membrane and consequently to an increase in calcium influx 15,16,29. Further investigations are needed to explain the mechanisms regulating TRPV5 and TRPV6 channels in parathyroid glands and their possible involvement in the tumourigenesis. On the other hand, various reports suggested that TRPV5 and TRPV6 channels may contribute to cell proliferation and malignant growth 7–12,30. Specifically, TRPV6 up-regulation was described in prostate cancer and other cancer of epithelial origin, such as breast, colon, thyroid and ovarian carcinomas 7. The link between tumour growth and protein overexpression of TRPV6 may involve the potentiation of calcium-dependent cell proliferation and the inhibition of apoptosis 12,30,31. In parathyroid adenomas, the neoplastic proliferation and the hormonal dysregulation, *i.e*. increased secretion of PTH, which in consequence leads to hypercalcaemia, are tightly linked 32. A shift in calcium-sensitive parathyroid hormonal regulation could be the cause or the result of abnormal parathyroid proliferation. Whether the TRPV5 and TRPV6 overexpression in this context could directly contribute to tumourigenesis or represent a secondary event of hypercalcaemia needs to be further elucidated. Moreover, besides the overexpression of TRPV5 and TRPV6, we observed a decrease of tumour suppressor protein p53 level in adenoma (data not shown). Generally, mRNA and proteins of TRPV5 and TRPV6 are up-regulated in tumour tissues and cell lines when compared with normal and correlate with tumour progression 8,31,33. Similarly, we observed an increase of protein expression of both channels in parathyroid adenomas in comparison with normal glands, but opposite results were obtained for mRNA quantitative expression. A decrease of TRPV5 and TRPV6 mRNAs, strictly correlated with vitamin D receptor (VDR) expression, was observed in renal cell carcinoma by Wu and co-workers 34. Indeed, a down-regulation of VDR mRNA is well documented in parathyroid adenomas 35. Nevertheless, the relationship between the expression of mRNA and its protein is not well characterized in human cancer. In fact, it has been described that discordant or opposite correlations can be observed, especially for proteins that present post-translational modifications leading to various isoforms and for less abundant proteins 36. This concept might explain the discordance we observed between mRNA and protein expression even if further investigations are needed.

All in all, our results show the presence of TRPV5 and TRPV6 channels in both normal parathyroid glands and parathyroid adenomas. Both channels were overexpressed in parathyroid adenomas. Our findings probably suggest that altered TRPV5 and TRPV6 expression might be associated with parathyroid adenoma. A limit to this study is the difficulty to obtain samples of both healthy or adenoma parathyroid and the absence of selective ligands for TRPV5 and TRPV6 vanilloid channels that makes difficult to perform conclusive functional experiments and to attribute calcium entry in parathyroid cells, only to these channels. On the other hand, until now, all the functional studies (electrophysiological and calcium imaging) were performed essentially on transfected cells 26,37,38. Two major limits occur in primary culture of parathyroid cells: first of all, the collection of the tissue is difficult, particularly normal gland; secondly, the time of the culture, because generally short-term cultures are obtained 39. In conclusion, further studies are needed to identify the mechanisms regulating TRPV5 and TRPV6 expression in parathyroid glands and their relation with the cell calcium signalling pathway and the pathological processes of these glands.
